# Telemedicine for rotator cuff syndrome: Asynchronous exercise and remote follow-up in a randomized controlled study

**DOI:** 10.1371/journal.pone.0344922

**Published:** 2026-03-31

**Authors:** Selkin Yilmaz Muluk, Nazlı Olcucu, Ozlem El

**Affiliations:** 1 Antalya Ataturk State Hospital, Department of Physical Medicine and Rehabilitation, Antalya, Turkey; 2 Dokuz Eylul University Hospital, Department of Physical Medicine and Rehabilitation, Izmir, Turkey; eCampus University: Universita degli Studi eCampus, ITALY

## Abstract

**Introduction:**

Rotator cuff syndrome (RCS) is a common musculoskeletal condition that requires rehabilitation. Telemedicine involving asynchronous exercise video and remote follow-up has emerged as a potential alternative to conventional in-person rehabilitation, offering advantages in accessibility and cost-effectiveness. This study aimed to evaluate the effectiveness of telemedicine compared to conventional methods in the rehabilitation of RCS.

**Methods:**

This randomized controlled trial was conducted at a public hospital physiatry clinic between November 2023 and June 2024. The telemedicine group (TG) received asynchronous exercise videos and remote video-call follow-ups; the control group (CG) received illustrated exercise brochures and in-person follow-ups. Outcomes were assessed at baseline, 15^th^ day, and 6^th^ month. The primary outcomes were pain (measured using the Visual Analog Scale, VAS) and functional levels (assessed using the Quick Disabilities of Arm, Shoulder, and Hand, QuickDASH). Secondary outcomes included exercise adherence, satisfaction, and travel burden. Data were analyzed using linear mixed-effects models.

**Results:**

Trial included 90 patients (mean age 51.19 ± 7.07 years), randomly assigned to TG (n = 45) or CG (n = 45). Both groups demonstrated within-group improvements in VAS (mean change −1.88 [95% CI −2.56 to −1.20]) and QuickDASH (−15.6 [95% CI −21.0 to −10.2]) scores from baseline to 15^th^ day (p < 0.001) and in VAS from baseline to 6^th^ month (−4.82 [95% CI −5.50 to −4.13], p < 0.001). No between-group differences were found in pain reduction at 15^th^ day (estimate = −0.27, 95% CI −1.44 to 0.89, p = 0.645) or at 6^th^ month (estimate = −0.40, 95% CI −1.57 to 0.77, p = 0.499), nor in functional improvement at 15^th^ day (estimate = −2.68, 95% CI −12.90 to 7.57, p = 0.607). The TG demonstrated higher exercise adherence (p = 0.027) and satisfaction (p < 0.001). Based on predefined minimal clinically important difference thresholds, all group differences fell within non-inferiority margins. The cost-effectiveness of telemedicine was 266.4 km and 1325.6 minutes saved per unit of VAS, and 11.34 km and 56.41 minutes saved per unit of QuickDASH improvement.

**Conclusion:**

Telemedicine is a viable alternative to traditional management of RCS, providing comparable pain relief, functional outcomes, and reduced travel burden. Future studies should assess long-term effects and broader populations.

## Introduction

The rotator cuff consists of the supraspinatus, infraspinatus, teres minor, and subscapularis muscles and tendons that encircle the shoulder joint. Rotator cuff syndrome (RCS) includes subacromial impingement, bursitis, tendonitis, and partial or complete tendon tears. These issues, caused by injury or degeneration, often result in shoulder pain [1]. The prevalence of shoulder pain varies widely across the globe, ranging from 0.67% to 55.2%, with higher rates observed in women. The annual incidence of shoulder pain is approximately 37.8 cases per 1000 people per year [2]. The prevalence increases with age, from 9.7% in those under 20–62% in individuals over 80 [3].

Reviews highlight exercise programs as a core recommendation for managing RCS, with acetaminophen, nonsteroidal anti-inflammatory drugs (NSAIDs), and corticosteroid injections also emphasized [4,5]. The primary goal in treating shoulder pain is to restore pain-free shoulder movement and function. A structured exercise program is as effective as surgery in subacromial pain syndrome, rotator cuff tendinopathy, partial-thickness tears, and atraumatic full-thickness tears [6]. Conservative treatment was shown to be effective in many patients, even with tears [7]. There is also a need to recommend avoiding heavy loads, repetitive motions, and overhead tasks [8–10].

Exercise programs, whether in a clinical setting or at home, effectively reduce pain and improve shoulder function in RCS [11]. Since supervised exercise provides no significant advantage except for adhesive capsulitis, home-based regimens can be recommended [12]. They should begin with range of motion and stretching exercises, progressing to strengthening exercises once mobility is restored [13–15]. Relying solely on brochures to perform exercises at home and attending regular clinical follow-ups presents two significant challenges. First, it’s not always possible to fully understand the exercises from visual illustrations alone, which can lead to incorrect or incomplete execution of the movements. Second, frequent hospital visits for follow-up care create an additional burden on healthcare systems and a significant time and cost burden for patients.

Telemedicine, a remote approach to assessment, treatment, and follow-up, driven by technological advancements and the COVID-19 pandemic, offers a viable alternative to traditional care. Asynchronous video-based exercises with remote physician follow-up can enhance exercise comprehension and reduce in-person visits. In randomized controlled non-inferiority trials, telemedicine-based rehabilitation programs were demonstrated to be non-inferior to traditional face-to-face rehabilitation in terms of clinical outcomes [16–18].

This study aimed to assess the effectiveness of a specific telemedicine model, utilizing asynchronous exercise videos and remote follow-ups for patients with RCS, comparing its outcomes to that of the traditional in-person method.

We hypothesized that a telemedicine-based exercise program would be non-inferior to standard in-person rehabilitation in reducing pain and improving function in patients with RCS. Non-inferiority was assessed based on minimal clinically important difference (MCID) thresholds previously established in the literature [19,20]. Secondary hypotheses included that telemedicine would show greater adherence and satisfaction, owing to increased accessibility and flexibility of care.

## Materials and methods

### Settings and participants

This parallel-design randomized controlled trial was conducted at a public hospital’s Physical Therapy and Rehabilitation Clinics. Participants were recruited over six months (started at 24/11/2023 and ended at 19/06/2024), and the trial concluded as scheduled. The trial was registered at ClinicalTrials.gov (NCT06194435) on 30 November 2023. Participant recruitment began on 24 November 2023, so registration was retrospective for 6 days. The delay occurred because the study protocol and institutional review board approval (23 November 2023) were completed first, and the institutional sign-off and ClinicalTrials.gov account activation/administrative steps required several business days to finalize. The authors confirm that all ongoing and related trials for this intervention are registered.

Ethical approval was obtained from Antalya Education and Research Hospital Ethical Committee (Ethical approval number: 16/28; date: 23/11/2023), and participants gave written informed consent per the Declaration of Helsinki. Data were archived on Zenodo (doi: 10.5281/zenodo.14655747), and the study followed CONSORT 2010 guidelines.

### Sample size calculation

The primary outcome for sample size estimation was pain intensity as visual analog scale (VAS). Based on an MCID of 1.2 points and SD of 2.0 from previous literature, a one-sided α = 0.05 and 80% power indicated a required sample of 35 participants per group for a non-inferiority design [16]. Accounting for an 8% dropout rate, the target sample was 38 per group (76 total). A parallel estimate for the Quick Disabilities of Arm, Shoulder, and Hand (QuickDASH) (MCID = 15.91, SD = 17.0) suggested a smaller required sample, so the VAS-based calculation was used for a more conservative design [20]. The non-inferiority margins were based on established MCID values, representing the smallest changes patients perceive as meaningful. These provided a clear and conservative clinical benchmark. Power was calculated for a two-group non-inferiority design without adjustment for secondary comparisons.

### Inclusion and exclusion criteria

Eligible participants had MRI-confirmed unilateral rotator cuff tendinopathy (tendinitis/ tendinosis), impingement, or partial rupture. All participants were required to be able to perform exercises, understand written materials, and use digital communication tools. Exclusion criteria included cognitive impairment, bilateral shoulder involvement, complete tendon rupture, shoulder instability, adhesive capsulitis, prior fractures or surgery on the same shoulder, instability, visual impairments, amputations, advanced cancer, uncontrolled diabetes, recent shoulder injections (≤6 months), prior physical therapy/home exercises (≤6 months), absence of digital device/internet access, or ongoing cancer treatment. No stopping guidelines or interim analyses were implemented.

### Interventions

On the first day, patients in both groups were evaluated face-to-face. They provided demographic data, reported their travel distance and time to the hospital, and completed baseline assessments, including VAS and QuickDASH. Then, on the same day, the participants of CG received an illustrated exercise brochure, and the participants of TG were provided with identical exercises as asynchronous video content to access remotely. The exercise program, adapted from the American Academy of Orthopaedic Surgeons’ guide, included range of motion and stretching exercises [21]. Before leaving the clinic, both groups were additionally instructed to avoid heavy lifting and repetitive movements. They also received an exercise diary to track three daily sessions for 15 days.

On days 7 and 15, CG had in-clinic follow-ups, while TG received remote follow-ups via video calls, which were via FaceTime or WhatsApp. During these follow-ups, the physician asked for pain status and exercise compliance, listened to patients’ concerns, and answered their questions about RCS and related exercises. Both in-person and remote patient-physician encounters were standardized to be identical, and no physical examination was performed during the in-person follow-up. Remote contacts were scheduled on days 7 and 15 via video calls (FaceTime/WhatsApp); asynchronous messaging was used ad hoc for clarifications. The typical clinician response time was within the same day (≤24 h).

After the 15^th^ day, all the participants were advised to continue the exercises and avoid heavy lifting. At the 6^th^ month, all participants’ VAS scores were reassessed via regular telephone calls. Participants in the TG had only one in-person hospital visit, as follow-ups were conducted remotely via video calls, compared to three visits in the control group.

### Outcome measurements

Primary outcomes included pain (VAS score, 0–10) and functional recovery (Quick-DASH). Secondary outcomes included exercise adherence (% of completed exercises), patient satisfaction (Likert scale, 1–5), and travel burden (distance in kilometers, time in minutes). Adherence was tracked with 15-day exercise diaries (three sessions/day). Participants in the TG sent photos of their diaries, while those in the CG brought their diaries to in-clinic visits. The QuickDASH tool assesses functionality, pain, symptoms, and quality of life through 11 questions on activities like gripping, carrying, and daily tasks. It has been validated, proven reliable, and adapted to the regional language [22,23]. The numeric VAS was used to express pain intensity on a scale from 0 (no pain) to 10 (worst pain). The 6^th^ month VAS assessment was added with ethics committee approval after protocol approval; no other changes were made to the methods or outcomes.

### Randomization and blinding

Participants were randomized using a computer-generated stratified sequence to balance age and sex. An independent clinical secretary, not involved in the study procedures, enrolled participants and assigned them according to the pre-generated sequence. Allocation concealment was maintained, as the randomization list was inaccessible to the investigators. For analysis, the secretary anonymized the allocation as Group A and Group B. Due to the nature of the intervention, blinding of participants and treating physicians was not feasible. Participants were randomly assigned by sex and age (30–49 or 50–64 years). Separate computer-generated block lists (block size 4) ensured balance within each stratum, and assignments were kept in numbered, sealed envelopes opened after baseline assessment.

### Statistical analysis

All analyses, including all randomized participants under an intention-to-treat (ITT) framework, were performed using IBM SPSS (v29.0.2.0) and Jamovi (v2.x, GAMLj). The normality of quantitative data was assessed using the Shapiro-Wilk test and visual inspection of Q-Q plots and histograms. Descriptive statistics were presented as means ± standard deviations for normally distributed continuous variables or medians (interquartile range, IQR) for non-normally distributed continuous variables, and as frequencies (%) for categorical variables. Baseline group differences were assessed with independent t-tests for normally distributed continuous data and Mann-Whitney U tests for non-normally distributed continuous data. Chi-square tests were used for categorical data comparisons.

Primary outcomes were analyzed using linear mixed-effects models with fixed effects for time, group, and time × group interaction, and a random intercept for each participant. Missing data were handled using restricted maximum likelihood assuming they were missing at random (MAR). The Satterthwaite approximation was used for degrees of freedom. Post hoc pairwise comparisons were Bonferroni-adjusted to control for Type I error. Since only three time points were compared (baseline, day 15, and month 6), the total number of within-group comparisons was limited to three per outcome, making Bonferroni a suitable and sufficiently conservative adjustment.

Effect sizes were calculated to interpret the magnitude of group differences. For between-group comparisons of normally distributed continuous variables, Cohen’s d was calculated. For non-parametric tests, effect size r was used. Cramér’s V was applied for categorical comparisons. Effect sizes were interpreted using standard thresholds: 0.2 (small), 0.5 (medium), and 0.8 (large) for Cohen’s d, 0.1 (small), 0.3 (medium), and 0.5 (large) for r and Cramér’s V. Travel and time efficiency of telemedicine was assessed based on travel distance and time saved per unit of improvement in VAS and QuickDASH scores. All outcomes other than the predefined primary measures (VAS and QuickDASH) were treated as exploratory and intended to generate hypotheses. Model fit was evaluated using residual diagnostics (QQ-plots and residuals vs fitted values) and found to be adequate. Assumption checks and supporting analyses are reported in Supplementary S1–S5 Tables.

## Results

### Descriptive statistics

**Fig 1:**
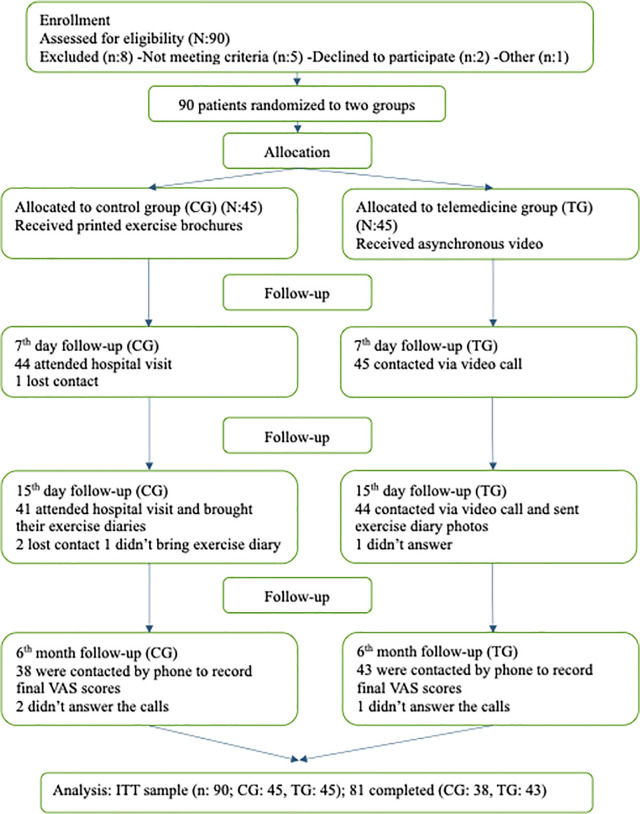
CONSORT flow diagram.

Although the target sample size was 76 total, 90 (aged 30–64 years) patients met eligibility criteria during the pre-specified enrollment period. Those patients with MRI-confirmed RCS were randomized in a 1:1 ratio to the telemedicine group (TG) or control group (CG). Following dropouts, 81 participants completed the study (Fig 1).

A total of 90 patients were randomized and included in the descriptive analysis. All inferential analyses, including linear mixed-effects modeling, were conducted using the ITT population (n = 90).

The mean age of participants was 51.19 ± 7.07 years. The cohort was predominantly female and married, with university being the most common education level. Less than half reported working in heavy-lifting jobs. Most had right shoulder involvement and were right-handed. In more than half of the cases, the affected shoulder was on the same side as the dominant hand. Nearly half of the patients (n:41) had tendinopathy, 25 had impingement accompanying tendinopathy, 24 had partial rupture on MRI. Baseline VAS and QuickDASH scores indicated moderate to severe pain and functional limitations. On average, participants traveled 7 km and spent 30 minutes per hospital visit. Full baseline characteristics are presented in Table 1.

**Table 1:** Descriptive characteristics of study participants.

Variable	Median (Q1, Q3) Mean ± SD (min, max)
Age	51.00 (46.00, 57.25)
Sex	female (54, 60%), male (36, 40%)
Marital status	married (72, 80%), single (18, 20%)
Education level	primary school (29, 32.2%), secondary school (6, 6.7%), high school (18, 20%), university (37, 41.1%)
Working conditions	lifting heavy (37, 41.1%), desk job (18, 20%), travelling (7, 7.8%), none (28, 31.1%)
Shoulder involved	right (53, 58.9%), left (37, 41.1%)
Dominant hand	right (83, 92.2%), left (7, 7.8%)
Drugs	NSAID (13, 14.4%), other painkillers (12, 13.3%), others (18, 20%), none (47, 52.2%)
VAS score	7.00 (5.00, 8.00)
QuickDASH score	50.95 ± 16.94 (17.50-88.64)
Distance to hospital (km)	6.9 (5.00, 8.05)
Time spent to reach the hospital (min)	30 (20.00, 40.50)

Note. CI: confidence interval. NSAID: nonsteroidal anti-inflammatory drugs. Other painkillers include paracetamol, gabapentin, tramadol, myorelaxants.

### Comparability of groups

At baseline, the TG and CG were largely comparable. No statistically significant differences were found for categorical variables (sex, marital status, working conditions, medication use) or continuous variables (age, VAS scores, QuickDASH), and effect sizes were small. Education level differed statistically between groups (p = 0.041), but the effect size was small (r = 0.22) with no meaningful median difference, indicating no clinically relevant imbalance. Overall, the groups were comparable for assessing treatment effects (Table 2).

**Table 2:** Baseline characteristics of control and telemedicine group.

Variable	Control group (n = 45)	Telemedicine group (n = 45)	p-value	Effect size
Age	50.00 (46.50, 55.00) years	54.00 (46.00,59.50) years	0.234	0.13
Sex	female (29, 64.4%) male (16, 35.6%)	female (25, 55.6%) male (20, 44.4%)	0.389	0.09
Marital status	married (36, 80%) single (9, 20%)	married (36, 80%) single (9, 20%)	1.000	0.00
Education level	4.00 (1.50, 4.00)	3.00 (1.00,4.00)	0.041	0.22
Working conditions	heavy-lifting (17, 37.8%), desk job (11, 24.4%), travelling (3, 6.7%), none (14, 31.1%)	heavy-lifting (20, 44.4%), desk job (7, 15.6%), travelling (4, 8.9%), none (14, 31.1%)	0.735	0.12
VAS score	7.00 (5.00, 8.00)	6.00 (5.00, 8.00)	0.883	0.02
QuickDASH score	53.52 ± 16.54 (95% CI:48.55–58.49)	48.37 ± 17.13 (95% CI:43.24–53.53)	0.152	0.31
Drug use	NSAID (6, 13.3%), other painkillers (8, 17.8%), others (10, 22.2%), none (21, 46.7%)	NSAID (7, 15.6%), other painkillers (4, 8.9%), others (8, 17.8%), none (26, 57.8%)	0.539	0.16

Note. NSAID: nonsteroidal anti-inflammatory drug. Other painkillers: paracetamol, gabapentin, tramadol, myorelaxants. Mann-Whitney U test is used for age, education level, VAS; independent samples t-test is used for QuickDASH; Chi-square test is used for others. Education level for CG: primary school (s) (24.4%), secondary s (4.4%), high s (20%), university (51.1%), for TG: primary s (40%), secondary s (8.9%), high s (20%), university (14, 31.1%).

### Linear mixed-effect models

Model assumptions were assessed prior to conducting linear mixed-effects modeling. Normality of residuals was evaluated using Q-Q plots and the Shapiro-Wilk test. Homoscedasticity was assessed via residual vs. fitted value plots, and linearity was verified through visual inspection of residual distributions. All assumptions were adequately met, supporting the robustness of the model results.

**Fig 2:**
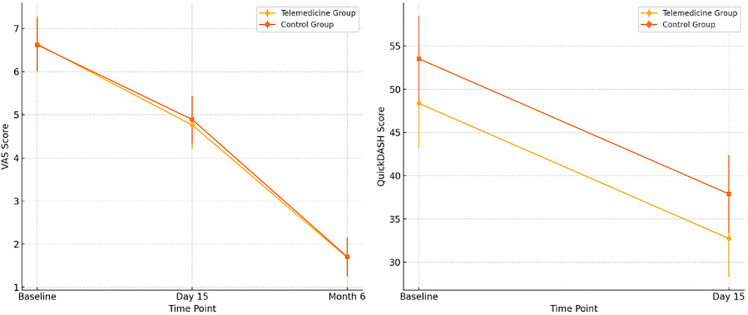
Estimated marginal means of VAS and QuickDASH scores over time by group.

Linear mixed-effects models showed significant improvements over time in both VAS (estimate = −1.88 [95% CI −2.56 to −1.20]) and QuickDASH (estimate = −15.62 [95% CI −21.00 to −10.21]) scores (p < 0.001) (Fig 2). However, neither the main effect of group nor the group × time interaction was significant, indicating that both groups improved similarly over time (VAS interaction at day 15: −0.27 [95% CI −1.44 to 0.89]; at month 6: −0.40 [95% CI −1.57 to 0.77]; QuickDASH interaction: −2.68 [95% CI −12.90 to 7.57]). Although the main effect of group was significant for QuickDASH (p = 0.015, Cohen’s d = 0.31), the absence of a significant interaction suggests that the trajectory of improvement was comparable across groups. Ranges for baseline–day 15 values and change scores are provided in S5 Table. Residual inspection confirmed adequate model fit. These findings support the clinical non-inferiority of the telemedicine intervention compared to the control group. Post hoc analyses confirmed significant reductions in VAS at all time points and in QuickDASH from baseline to day 15 (Tables 3,4).

**Table 3:** Linear mixed-effects model results for QuickDASH scores at baseline, and 15^th^ day.

Effect	Estimate	SE	95% CI (lower)	95% CI (upper)	df	t-statistic	p value
Intercept (baseline QuickDASH)	43.14	1.37	40.40	45.85	85.0	31.486	<0.001
Time (day 15 vs baseline)	−15.62	2.74	−21.00	−10.21	85.0	−5.701	<0.001
Group (telemedicine vs Control)	−6.47	2.60	−11.60	−1.35	84.7	−2.493	0.015
Time × group interaction	−2.68	5.19	−12.90	7.57	84.7	−0.516	0.607
Post hoc: day 15 vs baseline (Bonferroni)	−15.60	2.74	–	–	85.0	5.70	<0.001

Note. Time effects show within-group changes; group and interaction terms assess between-group differences. Estimates represent fixed effects from the linear mixed-effects model with random intercepts for subjects.

**Table 4:** Linear mixed-effects model results for VAS scores at baseline, 15^th^ day, and 6^th^ month.

Effect	Estimate	SE	95% CI Lower	95% CI Upper	df	t-statistic	p value
Intercept (baseline VAS)	4.402	0.143	4.120	4.683	121	30.820	<0.001
Time (day 15 vs baseline)	−1.880	0.347	−2.563	−1.197	120	−5.424	<0.001
Time (month 6 vs baseline)	−4.815	0.348	−5.500	−4.131	121	−13.851	<0.001
Group (telemedicine vs control)	−0.203	0.244	−0.683	0.277	120	−0.832	0.407
Time × group interaction (day 15)	−0.273	0.590	−1.436	0.890	118	−0.462	0.645
Time × group interaction (month 6)	−0.402	0.593	−1.570	0.765	119	−0.679	0.499
Post hoc: baseline vs day 15	1.88	0.347	1.19	2.57	120	5.42	<0.001
Post hoc: baseline vs month 6	4.82	0.348	4.13	5.50	121	13.85	<0.001
Post hoc: day 15 vs month 6	2.94	0.355	2.23	3.64	124	8.27	<0.001

Note. Time effects show within-group changes; group and interaction terms assess between-group differences. Estimates represent fixed effects from the linear mixed-effects model with random intercepts for subjects.

### Clinical non-inferiority assessment

Observed differences between groups were within established MCID thresholds, suggesting that the telemedicine intervention produced clinically comparable outcomes. These results indicate no clinically meaningful difference in pain or functional outcomes between the groups at either follow-up point (Table 5). All 95% confidence intervals fell entirely within the predefined MCID margins, confirming clinical non-inferiority. No adverse events, harms, or unintended effects were reported in either group throughout the study.

**Table 5:** Non-inferiority analysis based on median differences between groups.

Outcome	Hodges-Lehmann Estimate	95% CI for median difference	p-value	MCID	Conclusion
15th day VAS	0.00	−1.00 to 1.00	0.802	1.20	Non-inferior
15th day QuickDASH	4.55	−40.90 to 47.73	0.341	15.91	Non-inferior
6-month VAS	0.00	−6.00 to 6.00	0.606	1.20	Non-inferior

Note. Hodges-Lehmann estimates represent the median of all pairwise differences in change scores between the telemedicine and control groups. Confidence intervals were computed using non-parametric methods. The minimal clinically important difference (MCID) thresholds were predefined based on the literature: 1.20 for VAS and 15.91 for QuickDASH. care.

### Effect sizes

Cohen’s d effect sizes were calculated to quantify the magnitude of between-group differences. Values were 0.06 for short-term (15-day) VAS, 0.20 for long-term (6-month) VAS, and −0.15 for short-term QuickDASH. These small effect sizes indicate negligible differences between groups and further support the non-inferiority of the telemedicine intervention.

### Exercise adherence and patient satisfaction

The TG demonstrated significantly higher exercise adherence (mean rank = 47.62) than the control group (mean rank = 35.96), p = 0.027. Patient satisfaction was also significantly greater in the TG (mean rank = 52.24) compared to the CG (mean rank = 29.65), p < 0.001.

### Covariates and confounding

Because age, sex, baseline VAS, baseline QuickDASH, and other key characteristics were well balanced between groups (Table 2), no covariates were added to the primary mixed-effects models to avoid unnecessary over-adjustment.

### Travel and time efficiency of telemedicine

Participants in the TG made only one in-person hospital visit, compared to three visits in the CG. On average, TG participants traveled 6.15 km and spent 30.51 minutes for their single hospital visit. By avoiding two additional in-person follow-ups, they effectively saved 12.30 km of travel and 61.02 minutes of commuting time per person. Clinically, the TG showed a mean reduction of 4.95 points in pain intensity and an improvement of 17.47 points in functional status over the course of the study. When these outcome improvements are considered in relation to the travel and time saved, telemedicine yields an estimated reduction of 2.48 km and 12.32 minutes per unit improvement in VAS, and 0.70 km and 3.49 minutes per unit improvement in QuickDASH.

A compact summary of key between-group contrasts with 95% CIs and effect sizes is provided in Table 6.

**Table 6:** Key outcomes (TG vs CG): model-based between-group differences.

Outcome & timepoint	Between-group difference (estimate)	95% CI	p-value	Effect size
VAS, day 15 (Δ)	−0.27	−1.44 to 0.89	0.645	d = 0.06
VAS, month 6 (Δ)	−0.40	−1.57 to 0.77	0.499	d = 0.20
QuickDASH, day 15 (Δ)	−2.68	−12.90 to 7.57	0.607	d = −0.15
Adherence (%)	TG > CG	—	0.027	—
Satisfaction (1–5)	TG > CG	—	<0.001	—

**Note.** Δ represents the between-group difference in change from baseline (TG – CG) derived from the linear mixed-effects models. Negative values favor TG. *p* values for VAS and QuickDASH are from the mixed-effects models; those for adherence and satisfaction are from Mann–Whitney U tests. Cohen’s *d* represents the standardized effect size.

## Discussion

### Principal findings

This study showed that patients with rotator cuff problems reported high pain levels (VAS = 7) and considerable impairment in upper extremity function (QuickDASH = 50.95), underscoring the burden of the condition and the need for clinical care. It also found that telemedicine, utilizing asynchronous exercise videos and remote follow-ups, is a viable alternative to traditional in-person rehabilitation for patients with RCS.

Our primary findings showed that both the TG and the CG had significant within-group improvements in pain (VAS) and function (QuickDASH) from baseline to day 15, and continued improvements in pain (VAS) up to 6 months (p < 0.001). Crucially, the TG achieved pain reduction and functional improvement that was non-inferior to the conventional care group. However it is important to mention that the lack of significant group differences may partly reflect reduced power due to the conservative Bonferroni correction and simplified sample-size estimation. Additionally, the TG demonstrated significantly higher exercise adherence (p = 0.027) and patient satisfaction (p < 0.001).

To our knowledge, this is the first study to directly compare asynchronous tele-exercise with remote follow-up against brochure-based home exercise and in-clinic follow-up for RCS patients, while also documenting travel distance and time saved. Results of outcomes that were not primary were only exploratory.Comparison with previous studies

The study cohort had a mean age of 51 years, was female and had dominant hand involvement. The predominance of older age, female participants, and dominant hand involvement in our study aligns with previous literature [2,3,24].

Both groups showed significant short-term VAS and QuickDASH improvements, with sustained long-term pain relief, confirming the effectiveness of home exercises and practical recommendations. These findings align with previous studies [4,7,12]. A research has demonstrated that exercise reduced pain and disability in rotator cuff tendinopathy [25]. A study comparing a self-managed single-exercise program to standard physiotherapy reported comparable outcomes at 3, 6, and 12 months, supporting the idea that self-management can be as effective as supervised therapy [26].

Non-significant differences between groups and non-inferiority analysis in our study confirmed telemedicine as a scalable alternative to conventional care. These findings align with a previous research by Amarese AJ et al who reported that teleexercise studies using webcams, phone calls, videos, and smartphone apps have produced similar improvements in outcomes compared to clinic-based, in-person control interventions. These programs may be as effective as traditional exercise training without the burden of travel [27]. Likewise, a telerehabilitation study on shoulder pain found no major differences between fully remote digital physical therapy and conventional in-person therapy in QuickDASH scores, self-reported pain, surgery intent, analgesic use, mental health, engagement, or satisfaction [28]. Moreover, Huang et al. found telerehabilitation to be superior to home-based exercise in improving range of motion, functional outcomes, and quality of life for shoulder disorders, with even greater benefits when interventions lasted beyond 12 weeks [29]. Another meta-analysis also showed that telemedicine is superior to the conventional approach in terms of pain and range of motion [30].

An additional advantage of telemedicine in this study was its association with higher exercise adherence and patient satisfaction, likely due to the accessibility and clarity of exercise videos, which enhanced engagement and understanding. Literature shows mixed results on this topic; some studies report similar satisfaction and adherence between telemedicine and conventional methods, while others find higher adherence with telemedicine [31,32]. Asynchronous approaches have been shown to enhance adherence by offering clear demonstrations and reducing reliance on memory [31]. However, post-surgical patients often prefer in-person follow-ups, and telerehabilitation has not consistently demonstrated superior adherence in this group [33,34].

Beyond treatment efficacy, satisfaction, and adherence, telemedicine reduces travel burdens and addresses barriers such as transportation, long waiting times, financial constraints, and infection risks. Studies show these factors often limit access to in-person rehabilitation, especially for low-income and uninsured patients, leading to missed appointments and poorer health outcomes [35,36].

In our study, TG saved 26.64 km and 132.56 minutes per patient by reducing in-person visits. The cost-effectiveness ratio for telemedicine was 266.4 km and 1325.6 minutes saved per unit of VAS improvement and 11.34 km and 56.41 minutes per unit of QuickDASH improvement, confirming its ability to reduce logistical burdens while maintaining comparable clinical outcomes. These findings align with previous tele-epilepsy study of 3,698 virtual visits, which resulted in a total of 928,696 miles saved, with a median of 124.5 miles avoided per visit [37]. Similarly, a review of 5,695 telemedicine visits reported an average travel reduction of 145 miles and 142 minutes per visit, leading to an estimated annual cost savings of 18,555$ [38].

The findings of this study, including improvements in pain and function, higher adherence and satisfaction, and reductions in costs, environmental impact, and travel time, highlight telemedicine as a valuable addition to rehabilitation programs. Telerehabilitation appears to be as effective as in-person rehabilitation or superior to no rehabilitation for musculoskeletal, neurological, and cardiorespiratory disorders [32]. Its effectiveness extends to postoperative recovery, as a trial demonstrated that asynchronous telemedicine for knee and shoulder arthroplasty reduced visit times, enabled range of motion evaluations, and maintained high patient satisfaction [39]. Similarly, poststroke telerehabilitation was shown to be a viable alternative, particularly in underserved areas [40,41].

Moreover, telerehabilitation is equally effective as in-person care for respiratory and cardiac diseases, improving exercise capacity, quality of life, and breathing capacity [42,43]. Likewise, remote exercise sessions prevent falls, enhance physical function, adherence, and psychological well-being in elderly [44,45]. Since the COVID-19 pandemic, telerehabilitation has become increasingly widespread, demonstrating that it is effective, safe, and associated with high patient satisfaction [46–48].

Beyond rehabilitation, telemedicine has shown promise in other specialties by supporting neurological care, obesity management, and diabetes [49–53]. It also improves access to mental health, dermatology, obstetrics, and oncology services [54–58].

While telemedicine offers benefits, concerns remain over its rapid, unregulated growth during the COVID-19 pandemic. Challenges include quality of care, access disparities, privacy, safety, and ethical decision-making in non-clinical settings [59,60]. Although no adverse events or privacy issues were observed in our study, the lack of in-person supervision may have been a concern for patients needing closer monitoring.

### Limitations

Exercise adherence was based solely on patient reports; this may introduce reporting bias. More objective methods (e.g., wearable devices, digital sensors) can be used in future studies. The study was concluded at 6 months. The effectiveness, sustainability, and potential relapse rates of RCS could not be evaluated at longer-term follow-up.

Participants were a homogeneous group, aged 30–65, with unilateral shoulder problems. While this limits the generalizability of the results to other age groups or patients with bilateral shoulder problems, this allowed for more controlled analyses.

No objective measurements of shoulder range of motion, muscle strength, or ultrasound were performed during follow-up, but validated scales such as the VAS and QuickDASH were used. Additionally, the study was conducted in a single public hospital, limiting its generalizability to populations from different geographic regions with different socioeconomic or healthcare system conditions. Inclusion criteria included the ability to use a smartphone/tablet and access to the internet. This may create selection bias because it excludes patients who lack digital literacy or device access.

The sample size was based on a two-group design using MCID thresholds. Bonferroni correction for multiple comparisons was not included in the initial power estimate, so non-significant results should be interpreted with caution. Missing data were assumed to be missing at random; multiple imputation was not performed. Future studies could apply imputation or sensitivity analyses. Non-inferiority conclusions were guided mainly by MCID-based margins and 95% confidence intervals rather than p-values. Relatively small sample size may have reduced the ability to detect subtle differences. The variability in symptom duration among participants may also have introduced additional clinical heterogeneity.Due to the type of intervention, blinding of patients and physicians was not possible. Therefore, patient expectations and the researcher-patient interaction may have influenced treatment perception. However, statistical analyses were conducted independently and blinded. Finally, the study did not account for economic factors such as reimbursement policies, which may influence the real-world adoption of telemedicine models. No adverse events were reported in the study.

### Generalisability

The study can be generalized to individuals with uncomplicated rotator cuff syndrome who are middle-aged and older and have access to technology and can perform exercises at home. In these patients, asynchronous teleexercise and remote follow-up by a physician may be beneficial for pain reduction and functional gain.

### Clinical implications

Asynchronous video-based exercise with brief remote follow-ups can reduce travel and clinic load, making it suitable for public systems and underserved regions. The model requires only a smartphone and basic internet access, and it can be scaled up through shared libraries of standardized exercise videos combined with brief, scheduled remote consultations with clinicians.

## Conclusion

Asynchronous exercise video with remote follow-up is an effective alternative to conventional care for uncomplicated rotator cuff tendinopathy, impingement, and partial tears. It provides comparable pain relief and functional recovery while being cost and time-efficient. Future studies should assess long-term outcomes and broader applicability.

## Supporting information

S1 Table Tests of normality for baseline variables. Kolmogorov–Smirnov and Shapiro–Wilk test results for age, baseline QuickDASH, and baseline VAS.
(DOCX)

S2 Table Levene’s test (equal variances) and independent t-test for baseline QuickDASH. (DOCX)

S3 Table Kolmogorov–Smirnov and Shapiro–Wilk normality tests for key variables. (DOCX)

S4 Table Mann–Whitney U comparisons of change scores (VAS, QuickDASH), exercise adherence, and satisfaction between groups. (DOCX)

S5 Table Ranges for baseline and day-15 outcomes and change scores, with within-group (baseline vs day 15) and between-group comparisons. (.DOCX)
