# Supplementary material for: Telemedicine for rotator cuff syndrome: Asynchronous exercise and remote follow-up in a randomized controlled study
Source: PLoS One. 2026 Mar 31;21(3):e0344922. doi: 10.1371/journal.pone.0344922 (PMC13037984; doi:10.1371/journal.pone.0344922)
Supplement: S1 Table — Kolmogorov–Smirnov and Shapiro–Wilk test results for age, baseline QuickDASH, and baseline VAS. (DOCX) [file pone.0344922.s001.docx]

Supplementary Table 1 Tests of normality for variables

| Variable | Kolmogorov-Smirnov (Sig.) | Shapiro-Wilk (Sig.) | Normality |
| --- | --- | --- | --- |
| Age | 0.200 | 0.028 | Not normal |
| Initial QuickDASH score | 0.200 | 0.172 | Normal |
| Initial VAS score | <0.001 | 0.005 | Not normal |

This table provides the results of the Kolmogorov-Smirnov and Shapiro-Wilk tests for normality across three variables: age, initial QuickDASH, and initial VAS. Based on the significance values, **initial QuickDASH** follows a normal distribution (p > 0.05 for both tests), whereas **age**and **initial VAS** do not follow a normal distribution (p < 0.05 for Shapiro-Wilk).
