# Supplementary material for: Telemedicine for rotator cuff syndrome: Asynchronous exercise and remote follow-up in a randomized controlled study
Source: PLoS One. 2026 Mar 31;21(3):e0344922. doi: 10.1371/journal.pone.0344922 (PMC13037984; doi:10.1371/journal.pone.0344922)
Supplement: S2 Table — (DOCX) [file pone.0344922.s002.docx]

Supplementary Table 2 Test of equality variances for initial QuickDASH scores

| Test | Statistic | p value | Conclusion |
| --- | --- | --- | --- |
| Levene’s test (Equality of variances) | F = 0.041 | 0.840 | Variances are equal (p > 0.05). Use equal variances assumed t-test row. |
| t-test (Equal variances assumed) | t = 1.362 | 0.177 | No significant difference between group means (p > 0.05). |
| Mean difference | 5.09583 | - | One group's mean is 5.10 higher, but not statistically significant. |
| 95% Confidence interval | [-2.35, 12.54] | - | The confidence interval includes 0, indicating no significant difference. |

Table summarizes the independent samples t-test conducted for initial QuickDASH, which follows a normal distribution. The t-test results (t = 1.362, p = 0.177) indicate no statistically significant difference in initial DASH scores between the two groups. The mean difference between groups was 5.10, but the 95% confidence interval [-2.35, 12.54] includes 0, confirming the lack of significance. Levene’s test confirmed the equality of variances (p = 0.840), so the **equal variances assumed** t-test result was used.
