# Supplementary material for: Telemedicine for rotator cuff syndrome: Asynchronous exercise and remote follow-up in a randomized controlled study
Source: PLoS One. 2026 Mar 31;21(3):e0344922. doi: 10.1371/journal.pone.0344922 (PMC13037984; doi:10.1371/journal.pone.0344922)
Supplement: S3 Table — (DOCX) [file pone.0344922.s003.docx]

Supplementary Table 3 Tests of normality for key variables

| Variable | Test | Statistic | df | Sig. | Interpretation |
| --- | --- | --- | --- | --- | --- |
| Control Group |  |  |  |  |  |
| Difference in QuickDASH scores | Kolmogorov-Smirnov | 0.152 | 38 | 0.026 | Non-normal distribution |
|  | Shapiro-Wilk | 0.957 | 38 | 0.146 |  |
| Difference in VAS scores | Kolmogorov-Smirnov | 0.170 | 38 | 0.007 | Non-normal distribution |
|  | Shapiro-Wilk | 0.960 | 38 | 0.194 |  |
| Exercise adherence percentage | Kolmogorov-Smirnov | 0.131 | 38 | 0.100 | Normality assumed |
|  | Shapiro-Wilk | 0.952 | 38 | 0.104 |  |
| Telemedicine Group |  |  |  |  |  |
| Difference in QuickDASH scores | Kolmogorov-Smirnov | 0.080 | 43 | 0.200* | Normality assumed |
|  | Shapiro-Wilk | 0.986 | 43 | 0.863 |  |
| Difference in VAS scores | Kolmogorov-Smirnov | 0.163 | 43 | 0.006 | Non-normal distribution |
|  | Shapiro-Wilk | 0.928 | 43 | 0.010 |  |
| Exercise adherence percentage | Kolmogorov-Smirnov | 0.152 | 43 | 0.014 | Non-normal distribution |
|  | Shapiro-Wilk | 0.879 | 43 | <0.001 |  |

The Mann-Whitney U test was applied to compare groups due to non-normal distributions.
