# Supplementary material for: Telemedicine for rotator cuff syndrome: Asynchronous exercise and remote follow-up in a randomized controlled study
Source: PLoS One. 2026 Mar 31;21(3):e0344922. doi: 10.1371/journal.pone.0344922 (PMC13037984; doi:10.1371/journal.pone.0344922)
Supplement: S4 Table — (DOCX) [file pone.0344922.s004.docx]

Supplementary Table 4. Statistical comparison of clinical outcomes, exercise adherence, and satisfaction between control and telemedicine groups

| Variable | CG mean rank | TG mean rank | Mann-Whitney U | Z-Score | Asymp sig (2-tailed) | Interpretation |
| --- | --- | --- | --- | --- | --- | --- |
| Difference in QuickDASH scores | 38.36 | 43.34 | 716.500 | -0.951 | 0.341 | No significant difference |
| Difference in VAS scores | 41.89 | 40.21 | 783.000 | -0.328 | 0.743 | No significant difference |
| Exercise adherence percentage | 33.91 | 47.27 | 547.500 | -2.558 | 0.011* | Telemedicine group shows better adherence |
| Satisfaction | 29.24 | 51.40 | 370.000 | -4.642 | <0.001* | Telemedicine group shows higher satisfaction |

CG: control group, TG: telemedicine group. Asymp sig: asymptotic significance . *indicates significant difference. The Mann-Whitney U test revealed no significant differences between the groups in terms of the difference in QuickDASH (initial score-second score) and difference in VAS scores (initial score- second score). However, there was a significant difference in exercise adherence percentage between them (p = 0.011), with higher adherence observed in the telemedicine group. Furthermore, satisfaction levels were significantly higher in the telemedicine group compared to the control group (p < 0.001).
