# Supplementary material for: Telemedicine for rotator cuff syndrome: Asynchronous exercise and remote follow-up in a randomized controlled study
Source: PLoS One. 2026 Mar 31;21(3):e0344922. doi: 10.1371/journal.pone.0344922 (PMC13037984; doi:10.1371/journal.pone.0344922)
Supplement: S5 Table — (.DOCX) [file pone.0344922.s005.docx]

Supplementary Table 5. Ranges for within-group and between-groups comparisons between the baseline and 15^th^ day VAS, QuickDASH, 15^th^ day exercise adherence percentages, and satisfaction data.

|  | Control Group | |  | Telemedicine Group | |  | Control Group | Telemedicine Group |  |
| --- | --- | --- | --- | --- | --- | --- | --- | --- | --- |
| Variable | Baseline mean (r) | 15^th^ day mean (r) | p value (within-group) | Baseline mean (r) | 15^th^ day mean (r) | p value (within-group) | Difference mean (r) | Difference mean (r) | p value (between-group) |
| VAS score | 6.89 (3-10) | 4.84 (1-9) | <0.001 | 6.58 (2-10) | 4.63 (0-9) | <0.001 | 2.05 (-2-6) | 1.95 (-1-5) | 0.743 |
| QuickDASH score | 53.99 (20.45-81.82) | 38.87 (4.55-75) | <0.001 | 48.89 (17.50-88.64) | 31.42 (2.27-67.50) | <0.001 | 15.12 (-27.28-54.54) | 17.47 (-13.63-56.14) | 0.341 |
| Adherence % | - | 59.51 (20-100) | - | - | 72.60 (6.67-100) | - | - | - | 0.011 |
| Satisfaction | - | 3.95 (3-5) | - | - | 4.70 (3-5) | - | - | - | <0.001 |

Abbreviations: mean (r): mean (range), **– (dash):** not applicable (e.g., in "Exercise adherence," there is no baseline adherence to compare, and in "Satisfaction," it is assessed only post-intervention). NOTE. Difference values are baseline values minus 15^th^ day values.
